# Vascular plants dataset of the herbarium (HSS) of Agrarian Research Institute Finca “La Orden-Valdesequera” (CICYTEX), Extremadura, Spain

**DOI:** 10.3897/phytokeys.171.58900

**Published:** 2021-01-07

**Authors:** Francisco Márquez-García, David García-Alonso, María Josefa Guerra-Barrena, Francisco María Vázquez-Pardo

**Affiliations:** 1 Department of Forest Production and Vegetal Biodiversity, Institute of Agricultural Research “Finca La Orden- Valdesequera” (Cicytex), A5 km 372, 06187, Guadajira, Spain Institute of Agricultural Research “Finca La Orden- Valdesequera” Badajoz Spain

**Keywords:** Herbarium collection, HSS, Portugal, Southwest Iberian Peninsula, Spain, vascular plants

## Abstract

The HSS herbarium database includes 69,397 records of vascular plant taxa, representing 91.1% of the herbarium’s specimens as for December, 2019, which are available through the Global Biodiversity Information Facility (GBIF) website (accessible at https://doi.org/10.15468/siye1z). The database represents 4,343 species and 787 infraspecific taxa (530 subspecies, 130 varieties and 127 notho-species or hybrids) of 196 families and 1,164 genera, and 105 type sheets. So far, 97.7% of the databased records are georeferenced (geographic coordinates or MRGS coordinates) and the geographic area with the largest number of specimens is the southwest quadrant of the Iberian Peninsula (Spain and Portugal).

## The HSS herbarium

Forest biodiversity research (Vázquez et al. 1993) in the early 1990s by the Forest Production Department of the Agricultural Research Service (SIA) led to the creation of the HSS herbarium, which was initially located in the *Finca Santa Engracia* facilities (Badajoz, Extremadura, Spain). Later, 1995, the HSS herbarium was transferred to its current location, in the *Finca La Orden* (Guadajira, Badajoz), Institute of Agricultural Research *Finca La Orden-Valdesequera* of the Centre for Scientific and Technological Research of Extremadura (CICYTEX, Junta de Extremadura).

The HSS herbarium has five collections: fungi (HSS-F, 447 specimens), seeds (HSS-C, 727 entries), pollen (HSS-P, 402 entries), wood (HSS-X, 89 entries), and vascular plants (HSS, 76,136 specimens). Of the 76,136 total specimens, 91.1% (69,397 specimens) of the general vascular plant collection database is accessible on the GBIF platform (https://doi.org/10.15468/siye1z).

The vascular plant collection of the HSS herbarium is the result of research carried out over the last 25 years by the Department of Forest Production and Biodiversity. This research included studies on plant diversity (Vázquez et al. 2010; Vila-Viçosa et al. 2014) in the predominant forest systems of the southwest Iberian Peninsula, taxonomy, plants of ethnobotanical interest and their potential use as new crops, and ecosystem conservation centered on the study of endemic, rare or threatened species and the impact of potentially invasive species (Pinto-Gomes et al. 2006; Blanco and Vázquez 2014; Vázquez et al. 2015).

The main aim of this paper is to provide a vision about the specimens conserved in the HSS herbarium (diversity, distribution and types), and its potential uses in taxonomical, chorological and ecological studies.

## Taxonomic coverage

The HSS herbarium collection database contains 69,397 records belonging to 196 families, 1,164 genera, 4,343 species and 787 infraspecific taxa (530 subspecies, 130 varieties, and 127 notho-species or hybrids). Of the specimens in the collection 98.5% are identified at species level.

97.4% of the specimens housed in the HSS herbarium database are angiosperms (Magnoliophyta Cronquist, Takht. & Zimmerm. *ex* Reveal) with thirteen groups/clade based in APGIII (2009), APGIV (2016), Chase and Reveal (2009), and Reveal and Chase (2011). 2.0% of the specimens are ferns (Pteridophyta Haeckel) with four subclasses based in Christenhusz et al. (2011a): Equisetidae Warm., Lycopodiidae Bek., Ophioglossidae Klinge, Polypodiidae Cronquist, Takht. & Zimmerm. Finally, 0.6% are gymnosperms (Coniferophyta W.Zimm.) distributed in three subclasses based in Christenhusz et al. (2011b): Ginkgoidae Engl., Pinidae Cronquist, Takht. & Zemmerm, and Gnetidae Pax (Table 1) (See Suppl. material 1: Taxonomic coverage of the HSS Herbarium).

**Table 1.** Taxonomic coverage of the HSS Herbarium.

	Clade					Subclass	Specimens number	% value
Ferns	Ferns					Equisetidae	73	0.1
						Ophioglossidae	23	0.03
						Polypodiidae	1,168	1.7
	Lycophytes					Lycopodiidae	148	0.2
Gymnosperms						Ginkgoidae	1	0
						Gnetidae	12	0.02
						Pinidade	415	0.6
Angiosperms	Basal angiosperms						37	0.05
	Mesangiospermae	Magnoliids					280	0.4
		Monocots					16,994	24.5
		Eudicots					1,767	2.6
			Superasterids				4,159	6.0
				Asterids			946	1.4
					Campanulids		10,021	14.4
					Lamiids		10,169	14.6
			Superrosids				706	1.0
				Rosids			737	1.1
					Fabids		15,984	23.0
					Malvids		5,753	8.3
		Probable sister of Eudicots					4	0.01

**Figure 1.**
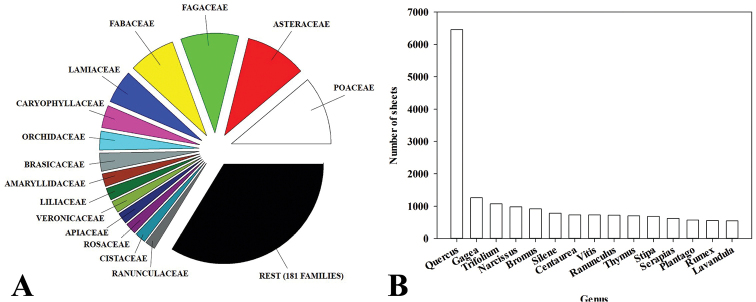
Families with greatest number of specimens in the HSS Herbarium (**A**) Genera represented by the highest number of specimens in the HSS Herbarium (**B**).

The ten families with the highest number of specimens are: Poaceae Barnhart (7,728 specimens), Asteraceae Bercht. & J.Presl (6,945 specimens), Fagaceae Dumort. (6,541 specimens), Fabaceae Lindl. (5,277 specimens), Lamiaceae Martinov (3,763 specimens), Caryophyllaceae Juss. (2,528 specimens), Orchidaceae Juss. (2,117 specimens) Brassicaceae Burnett (2,031 specimens), Amaryllidaceae J.St.-Hil. (1,444 specimens), and Liliaceae Juss. (1,372 specimens). Ten genera with the largest number of specimens are: *Quercus* L. (6,454 specimens), *Gagea* Salisb.(1,261 specimens), *Trifolium* L. (1,071 specimens), *Narcissus* L. (978 specimens), *Bromus* L. (918 specimens), *Silene* L. (781 specimens), *Centaurea* L. (731 specimens), *Vitis* L. (730 specimens), *Ranunculus* L. (725 specimens), and *Thymus* L. (703 specimens) (Fig. 1).

**Figure 2.**
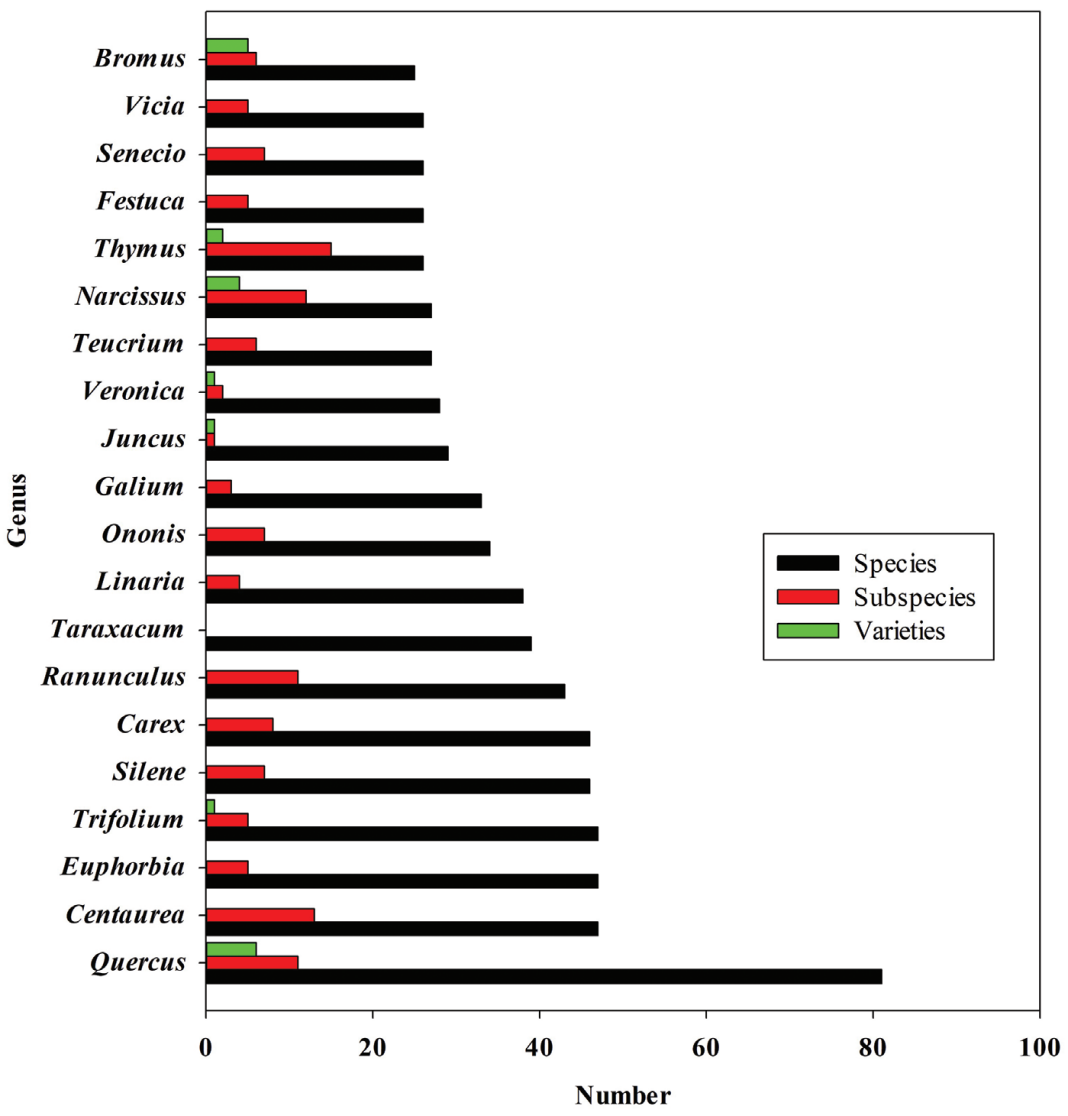
Genera with greatest number of species and infraespecific taxa (subspecies and varieties) in the HSS Herbarium.

Regarding the genera, those with the greatest number of species and infraespecific taxa (subspecies and varieties) represented are *Quercus* (81 species, 13 subspecies and 45 hybrids), *Centaurea* (47 species and 13 subspecies), *Euphorbia* L. (47 species and 5 subspecies), and *Trifolium* (47 species and 5 subspecies) (Fig. 2).

## Geographical coverage

The geographical data are structured in the specimens on three levels: geographical coordinates (longitude, latitude, WGS84 datum), MGRS (Military Grid Reference System) coordinates with an accuracy of 10,000 or 1,000 meters (ETRS89 datum) and locality assignment indicating the continent, country, province, municipality and town.

The HSS herbarium database only includes 60 records without continent or country data, 244 records without province data and 466 records without municipality or town data. 40% of the records are georeferenced with geographic coordinates and MRGS coordinates with precision of 1,000 meters, 57.7% of the records are georeferenced with MRGS coordinate assignment with precision of 10.000 meters, and only 2.3% of the records lack coordinates.

The geographical distribution of the materials preserved in the HSS herbarium is concentrated in the European continent (67,914 specimens, 97.8%), with small collections from other continents: Africa (959 specimens, 1.3%), North America (432 specimens, 0.6%), South America (47 specimens, 0.07%), Asia (25 specimens, 0.04%), and Oceania (20 specimens, 0.03%).

**Figure 3.**
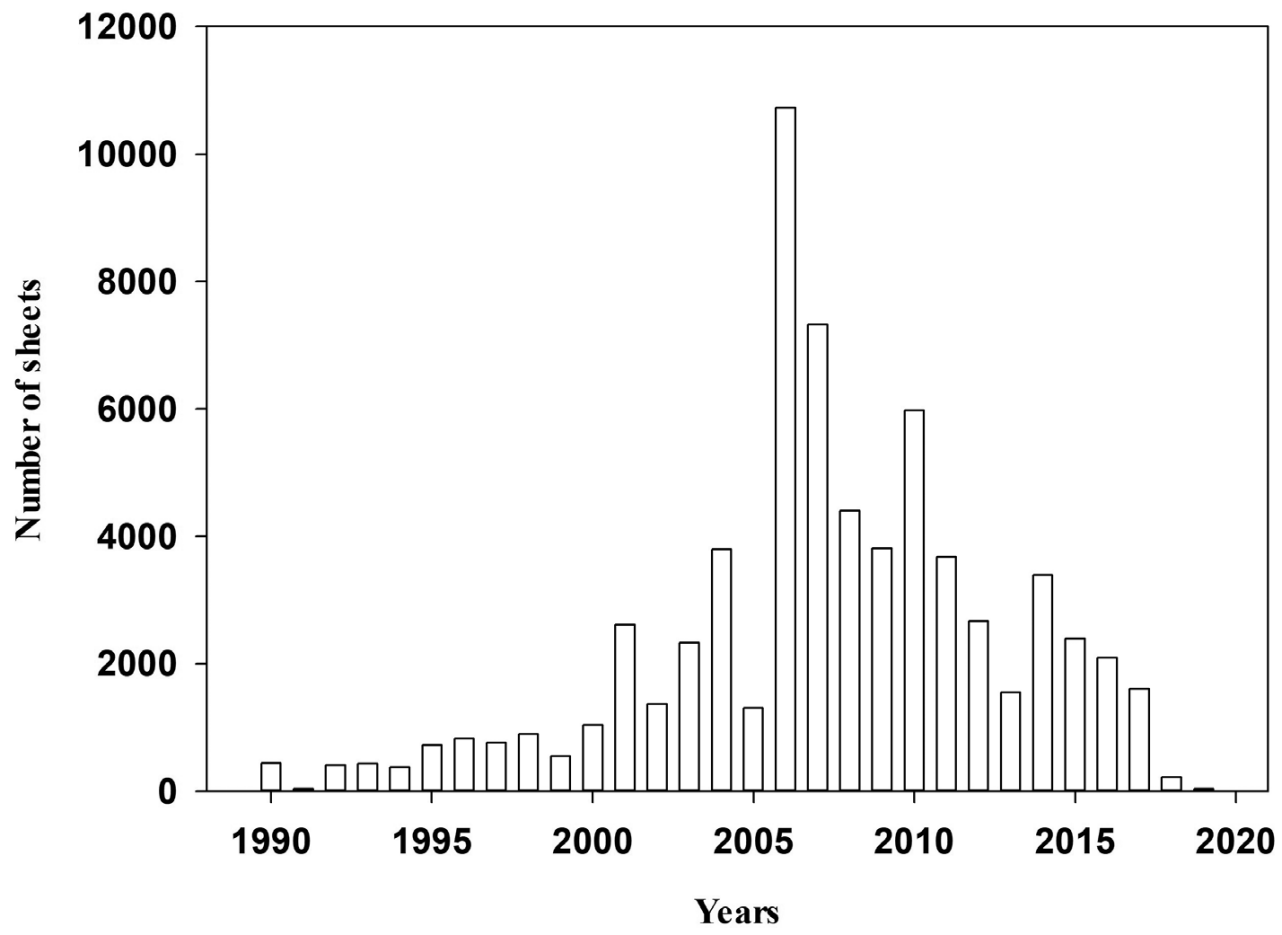
Geographical distribution of specimens of the HSS Herbarium in the Iberian Peninsula.

The geographical area with the highest number of specimens in the HSS Herbarium is the southwest quadrant of the Iberian Peninsula, which includes the Spanish provinces of Badajoz (27,542 records), Cáceres (20,727 records), Ávila (1,165 records), Salamanca (942 records), Huelva (966 records) and Seville (310 records), and the Portuguese provinces of High Alentejo (2,339 records), Low Alentejo (2,225 records), Algarve (1,112 records), and Estremadura (615 records) (Fig. 3).

In addition, there is notable representation from North Africa [Morocco (748 records) and Tunisia (209 records)], linked to collection trips for the study of the flora of the Atlas Mountains, being important in the description of new species and subspecies (Vázquez and Devesa 1997; Vázquez and Ramos 2007; Vázquez et al. 2012).

## Temporal coverage

The HSS herbarium database includes specimens from 1906 to 2019, distributed in two periods: before 1990 the collection was growing from exchanges and donations from private herbaria (1,353 specimens), and after 1990 it was growing due to floristics and research activities (67,813 specimens). Finally, there are 231 specimens without collection data.

**Figure 4.**
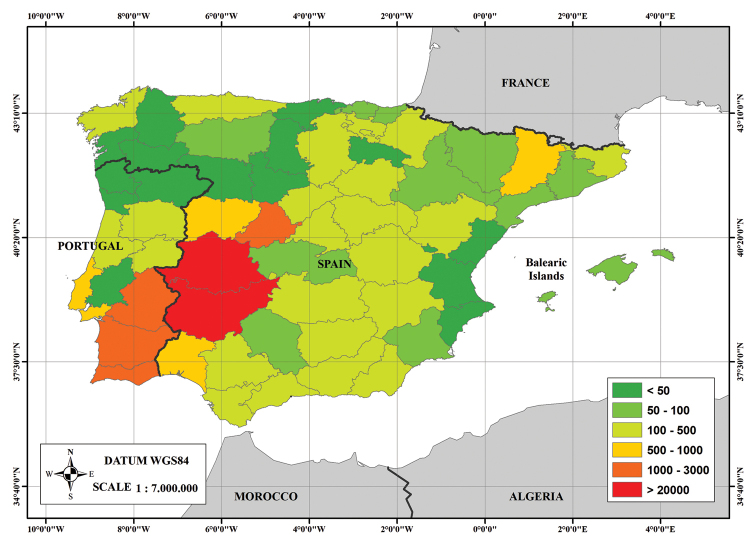
Number of specimens collected between 1990 and 2020.

Between 1990 and 2019, the period of greatest activity and growth in the collection are the five-year period 2006–2010, with collections of more than 3,500 specimens per year, linked to the study of the unique and threatened flora and the state of conservation of the predominant habitats in Extremadura (Palacios et al. 2010) (Fig. 4).

The monthly distribution of the specimens preserved in the HSS herbarium shows that the months with the highest collection activity are March, April, May and June corresponding to spring in the northern hemisphere.

## Plant processing procedures

The methodology used at the HSS herbarium for preserving specimens involves pressing and drying the fresh materials. To do this, the fresh material is placed between sheets of blotting paper and thick cardboard, including a sheet of corrugated aluminum foil for every 10–15 specimens, to facilitate drying (Singh and Subramaniam 2008). Drying is done at room temperature, with hot air used in exceptional cases. After pressing and drying, the material is subjected to freezing (-40 °C) for 48 hours to facilitate the conservation of the material, thus avoiding insect or fungal attack. This process (adapted from Forman and Bridson 1998) is repeated every 8–12 months until the specimens are accessioned.

## Quality control

1. Classification and identification phase. For the inclusion of plant material in the HSS herbarium, taxonomic identification is required, including assignment to genus, species, or to an infraspecific level where necessary. Works used in the identification process are referenced. For peninsular flora, the works of Amaral (1971–2003), Castroviejo (1986–2019), Devesa (1995), Tutin et al. (1964–1980), Valdés et al. (1987) are used. In addition, the determination of synonyms and taxonomic authors are consulted in the databases of The Plant List (http://www.theplantlist.org/), IPNI (The International Plant Names Index, http://www.ipni.org/), World Checklist of Selected Plant Families (WSSP) (https://wcsp.science.kew.org/), and the Euro+Med PlantBase (the information resource for Euro-Mediterranean plant diversity, http://ww2.bgbm.org/EuroPlusMed/). Finally, the organization of materials is done following the latest research on phylogeny of the plant kingdom: APGIII (2009), APGIV (2016), Chase and Reveal (2009), and Reveal and Chase (2011) in angiosperms; Christenhusz et al. (2011a); Pryer et al. (2004), and Smith et al. (2006) in ferns; and Christenhusz et al. (2011b) in conifers.
2. Georeferencing process. Approximately 98% of the specimens preserved in the HSS herbarium contain information regarding coordinates (UTM or geographic), with different levels of accuracy. There are 40% (27,761 specimens) with geographic coordinates and a level of uncertainty lower than 100 m. The working methodology followed for the georeferencing of the new data has been based on the collection of geographical coordinates (WGS84) with GPS in the field of sampling and collection points. Subsequently, at the laboratory, using web geoportals (https://www.ign.es/iberpix2/visor/, for Spain and http://geoportal.lneg.pt/geoportal/mapas/index.html for Portugal), province, municipality, town and altitude are assigned to each new record. For older specimens which did not have coordinates recorded in the field, the GEOLocate application (Rios and Bart 2010), and web geoportals, previously indicated, are used to assign geographic coordinates. Finally, with the help of geographic information systems such as QGIS (https://qgis.org/en/site/) and shapefile layers of MGRS coordinates (precise to 1 km2) obtained from the National Geospatial-Intelligence Agency (USA) (https://earth-info.nga.mil/), the UTM1x1 coordinates of the collection point are assigned.
3. Computerization and web publication (GBIF). The database has Access support following Darwin Core standards (https://dwc.tdwg.org/) on biological biodiversity, and the data are periodically reviewed using OpenRefine (https://openrefine.org/). For the publication of the HSS database in the GBIF portal, the Integrated Publishing Toolkit (IPT) portal of the Spanish GBIF network (https://ipt.gbif.es/resource?r=hss) is used.

## Storage

The herbarium storage room is equipped with humidity and temperature control (30% humidity and 10 °C temperature), hermetically sealed mobile shelves and cardboard boxes. The specimens are organized into four groups (ferns, conifers, angiosperms-monocots and other angiosperms). Within each group specimens are ordered alphabetically following the sequence: families, genera, species, subspecies, varieties, and forms.

## Interest in and use of the collection

The HSS herbarium includes 4560 taxa collected in the Iberian Peninsula, excluding hybrids, which represents around 40% of the 11,500 estimated taxa known from the Iberian flora, according to Flora Iberica (Castroviejo 1986–2019) and the web portals GBIF (https://www.gbif.org/), The Plant List (http://www.theplantlist.org/), and Euro + Med PlantBase (https://www.emplantbase.org/).

**Figure 5.**
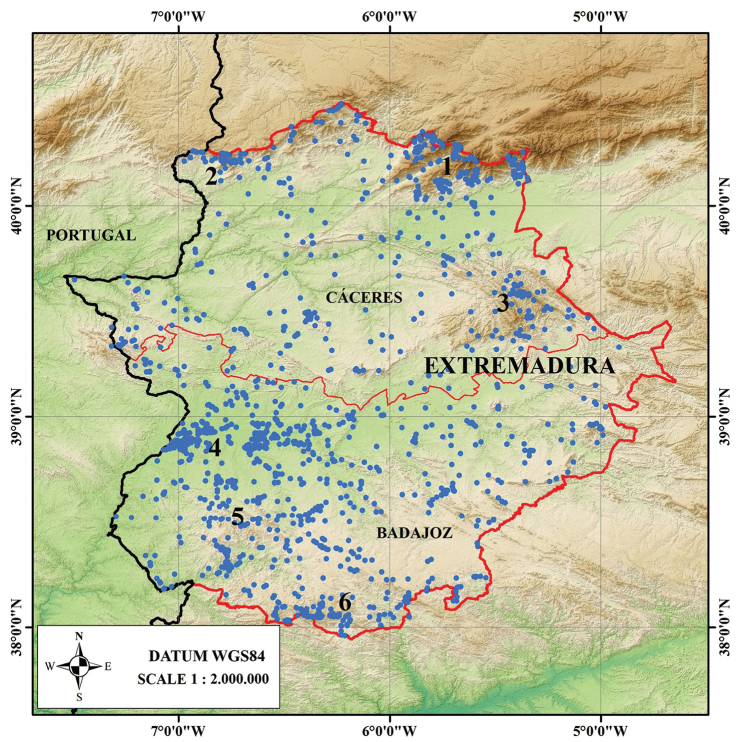
Collection points of the HSS Herbarium in Extremadura (Note: **1** southern slope of the Gredos Mountain range **2** Gata Mountain range **3** Villuercas Mountain range **4** Guadiana River valley **5** Badajoz Mountain range **6** foothills of Sierra Morena).

Regarding the flora of Extremadura, the herbarium holds specimens of 2986 taxa and 113 hybrids, approximately 98% of the taxa known from Extremadura (Castroviejo 1986–2019; Devesa 1995). In addition, the areas with the densest plant collections are located in protected areas with the highest plant diversity and best preserved in the region. These areas include the southern slope of the Gredos Mountain range, Gata Mountain range, Villuercas Mountain range, Badajoz Mountain range, the foothills of Sierra Morena, and Guadiana River valley (Fig. 5).

The HSS collection of vascular plants has served as the basis for various scientific works on the flora and vegetation of Extremadura and the bordering territories of Spain and Portugal. Among them are: the reviews and studies of the family Orchidaceae (Vázquez and Ramos 2005; Vázquez 2007, 2008a, 2008b, 2009; Vázquez et al. 2012), the genus *Quercus* (Vázquez et al. 1993, 2004; Vázquez 1995; Vázquez and Coombes 2016), *Stipa* L. (Vázquez and Devesa 1997; Vázquez and Ramos 2007), *Narcissus* (Vázquez et al. 2009; Vázquez 2013), *Bromus* (Vázquez and Scholz 2008), *Thymus* (Blanco et al. 2007), *Thymbra* L. (Blanco et al. 2007), *Scolymus* Tourn *ex* L. (Vázquez 2000), *Typha* Tourn *ex* L. (Vázquez 2012), *Taraxacum* F.H.Wigg (Vázquez 2014), *Festuca* L. (Vázquez and García 2016), *Vitis* L. (Vázquez and García 2017), and *Callitriche* L. (Márquez et al. 2017)

In addition, the HSS herbarium actively participates in the “Flora Iberica” project by providing material for the study of various genera of the Iberian Peninsula and Balearic Islands. The work carried out in the HSS herbarium allowed for the creation, in 2007, of the scientific journal “Folia Botanica Extremadurensis” a journal dedicated to scientific works and studies on the flora and vegetation of the southwest Iberian Peninsula.

Finally, the HSS herbarium contains 105 type sheets (82 holotypes, 12 isotypes, 9 paratypes, 1 isoparatype, and 1 neotype) (See Suppl. material 2: Types of the HSS Herbarium). The families with largest number of type sheets are Orchidaceae [45 type sheets, of seven genera: *Ophrys* L. (19), *Orchis* Tourn. *ex* L. (10), *Neotinea* Rchb.f. (4), *Anacamptis* Rich. (5), *Serapias* L. (5), *Dactylorhiza* Neck. *ex* Nevski (1), and ×*Cephalorchis* F.M.Vázquez (1)], Poaceae [19 type sheets, of seven genera: *Celtica* F.M.Vázquez & Barkworth (5), *Stipa* (5), *Bromus* (3), *Poa* L. (3), *Alopecurus* L. (1), *Festuca* (1), and *Helictochloa* Romero Zarco (1)], Fagaceae (15 type sheets, genus *Quercus*), and Amaryllidaceae (10 type sheets, genus *Narcissus*). All type sheets in the HSS herbarium are from Spain (89 type sheets, 77 of them from Extremadura), Portugal (10 type sheets), Morocco (5 type sheets), and Tunisia (1 type sheets).

## Maintenance and future work

Currently, the HSS herbarium has more than 6,000 sheets, corresponding to the collection trips of 2018–2019, which are not included in the database. The most immediate work is focused updating the database to include these records, and its subsequent updating in the GBIF network. Digitisation of the collection is currently prioritized as well.

Finally, the maintenance of the collection represents processing between 2,000–3,000 specimens annually.

## Dataset description

**Object name**: Darwin Core Archive (DwC-A) Herbario HSS Finca La Orden-Valdesequera (CICYTEX). Gobierno de Extremadura

**Collection Identifier**: 837acfc2-f762-11e1-a439-00145eb45e9a

**Character encoding**: UTF-8

**Format name**: Darwin Core Archive format

**Format version**: 1.7

**Distribution**: https://doi: 10.15468/siye1z

**Publication date of data**: 2020-06-25

**Language**: Spanish

**Licences of use**: Creative Commons Attribution Non-Commercial (CC-BY-NC) 4.0 License

**Metadata language**: Spanish

**Date of metadata creation**: 2015-03-16

**Hierarchy level**: Dataset

## Supplementary materials

Supplementary material 1 Taxonomic coverage of the HSS Herbarium Data type Taxonomic rank https://binary.pensoft.net/file/494927This dataset is made available under the Open Database License (http://opendatacommons.org/licenses/odbl/1.0/). The Open Database License (ODbL) is a license agreement intended to allow users to freely share, modify, and use this Dataset while maintaining this same freedom for others, provided that the original source and author(s) are credited.Francisco Márquez-García, David García-Alonso, María Josefa Guerra-Barrena, Francisco María Vázquez-Pardo

Supplementary material 2 Types HSS Herbarium Data type occurrence https://binary.pensoft.net/file/494928This dataset is made available under the Open Database License (http://opendatacommons.org/licenses/odbl/1.0/). The Open Database License (ODbL) is a license agreement intended to allow users to freely share, modify, and use this Dataset while maintaining this same freedom for others, provided that the original source and author(s) are credited.Francisco Márquez-García, David García-Alonso, María Josefa Guerra-Barrena, Francisco María Vázquez-Pardo
