# Supplementary material for: Vascular plants dataset of the herbarium (HSS) of Agrarian Research Institute Finca “La Orden-Valdesequera” (CICYTEX), Extremadura, Spain
Source: PhytoKeys. 2021 Jan 7;171:47–59. doi: 10.3897/phytokeys.171.58900 (PMC7809011; doi:10.3897/phytokeys.171.58900)
Supplement: Supplementary material 1 — Taxonomic coverage of the HSS Herbarium [file phytokeys-171-047-s001.doc]

**Taxonomic ranks**

**Kingdom:** Plantae Copeland.

**Subclasses (in gymnosperm and ferm):** Equisetidae, Ginkgosidae, Gnetidae, Lycopodiidae, Ophioglossidae, Pinidae, Polypodiidae.

**Order:** Alismatales R.Br. *ex* Bercht. & J.Presl, Apiales Nakai, Aquifoliales Senft, Arecales Bromhead, Asparagales Link, Asterales Link, Araucariales Henkel & W.Hochst., Berberidopsidales Doweld, Boraginales Juss. *ex* Bercht. & J.Presl, Brassicales Bromhead, Buxales Takht. *ex* Reveal, Caryophyllales Juss. *ex* Bercht. & J.Presl, Celastrales Link, Ceratophyllales Link, Commelinales Mirb. *ex* Bercht. & J.Presl, Cornales Link, Crossosomatales Takht. *ex* Reveal, Cucurbitales Juss. ex Bercht. & J.Presl, Cupressales Link, Cyatheales A.B.Frank, Dioscoreales Mart., Dipsacales Juss. *ex* Bercht. & J.Presl, Ephedrales Dumort, Equisetales DC. *ex* Bercht. & J. Presl, Ericales Bercht. & J.Presl, Fabales Bromhead, Fagales Engl., Garryales Mart., Gentianales Juss. *ex* Bercht. & J.Presl, Geraniales Juss. ex Bercht. & J.Presl, Ginkgoales Gorozh, Hymenophyllales A.B.Frank, Isoetales Prantl, Lamiales Bromhead, Laurales Juss. *ex* Bercht. & J.Presl, Liliales Perleb, Lycopodiales DC. *ex* Bercht. & J.Presl, Magnoliales Juss. *ex* Bercht. & J.Presl, Malpighiales Juss. *ex* Bercht. & J.Presl, Malvales Juss. *ex* Bercht. & J.Presl, Myrtales Juss. *ex* Bercht. & J.Presl, Nymphaeales Salisb. *ex* Bercht. & J.Presl, Ophioglossales Link, Osmundales Link, Oxalidales Bercht. & J.Presl, Pinales Gorozh, Piperales Bercht. & J.Presl, Poales Small, Polypodiales Bercht. & J. Presl, Proteales Juss. *ex* Bercht. & J.Presl, Psilotales Prantl, Ranunculales Juss. *ex* Bercht. & J.Presl, Rosales Bercht. & J.Presl, Salviniales Link, Santalales R.Br. *ex* Bercht. & J.Presl, Sapindales Juss. *ex* Bercht. & J.Presl, Saxifragales Bercht. & J.Presl, Selaginellales Prantl, Solanales Juss. *ex* Bercht. & J.Presl, Vitales Juss. *ex* Bercht. & J.Presl, Zingiberales Griseb., Zygophyllales Link.

**Families:** Acanthaceae Juss., Aceraceae Juss., Actinidiaceae Engl. & Gilg, Adoxaceae E.Mey., Aextoxicaceae Engl. & Gilg, Aizoaceae Martinov, Alismataceae Vent., Amaranthaceae Juss., Amaryllidaceae, Anacardiaceae R.Br., Annonaceae Juss., Apiaceae Lindl., Apocynaceae Juss., Aquifoliaceae Bercht. & J.Presl, Araceae Juss., Araliaceae Juss., Araucariaceae Henkel & W.Hochst., Arecaceae Bercht. & J.Presl, Aristolochiaceae Juss., Asclepiadaceae Borkh., Asparagaceae Juss., Aspleniaceae Newman, Asteraceae, Athyriaceae Alston, Balsaminaceae A.Rich., Berberidaceae Juss., Betulaceae Gray, Bignoniaceae Juss., Blechnaceae Newman, Boraginaceae Juss., Brassicaceae, Bromeliaceae Juss., Butomaceae Mirb., Buxaceae Dumort., Cactaceae Juss., Callitrichaceae Link, Campanulaceae Juss., Cannabaceae Martinov, Cannaceae Juss., Capparaceae Juss., Caprifoliaceae Juss., Caryophyllaceae, Casuarinaceae R.Br., Celastraceae R.Br., Ceratophyllaceae Gray, Chenopodiaceae Vent., Cistaceae Juss., Cleomaceae Horan., Clusiaceae Lindl., Colchicaceae DC., Commelinaceae Mirb., Convallariaceae Horan., Convolvulaceae Juss., Coriariaceae DC., Cornaceae Bercht. & J.Presl,, Crassulaceae J.St.-Hil., Cryptogrammaceae Pic.Serm., Cucurbitaceae Juss., Culcitaceae Pic.Serm., Cupressaceae Gray, Cymodoceaceae Vines, Cyperaceae Juss., Cystopteridaceae (Payer) Shmakov, Davalliaceae M.R.Schomb., Dennstaedtiaceae Pic.Serm., Dioscoreaceae R.Br., Dipsacaceae Juss., Droseraceae Salisb., Dryopteridaceae Herter, Ebenaceae Gürke, Elaeagnaceae Juss., Elatinaceae Dumort., Empetraceae Hook. & Lindl., Ephedraceae Dumort., Equisetaceae Michx.*ex* DC., Ericaceae Juss., Euphorbiaceae Juss., Fabaceae, Fagaceae, Frankeniaceae Desv., Garryaceae Lindl., Gentianaceae Juss., Geraniaceae Juss., Gesneriaceae Dumort., Ginkgoaceae Engl., Globulariaceae DC., Grossulariaceae DC., Haloragaceae R.Br., Hamamelidaceae R.Br., Hippocastanaceae A.Rich., Hydrangeaceae Dumort., Hydrocharitaceae Juss., Hymenophyllaceae Mart., Hypoxidaceae R.Br., Iridaceae Juss., Isoetaceae Dumort., Juglandaceae DC. *ex* Perleb, Juncaceae Juss., Juncaginaceae Juss., Lamiaceae, Lauraceae Juss., Lemnaceae Gray, Lentibulariaceae Rich., Liliaceae, Linaceae DC. *ex* Perleb, Lycopodiaceae P.Beauv. *ex* Mirb., Lythraceae J.St.-Hil., Magnoliaceae Juss., Malvaceae Juss., Marsileaceae Mirb., Martyniaceae Horan., Melanthiaceae Batsch *ex* Borkh., Melastomataceae Juss., Meliaceae Juss., Menyanthaceae Dumort., Molluginaceae Bartl., Monimiaceae Juss., Monotropaceae Nutt., Moraceae Gaudich., Musaceae Juss., Myoporaceae R.Br., Myrtaceae Juss., Nyctaginaceae Juss., Nymphaeaceae Salisb., Oleaceae Hoffmanns. & Link, Onagraceae Juss., Ophioglossaceae Martinov, Orchidaceae, Orobanchaceae Vent., Osmundaceae Martinov, Oxalidaceae R.Br., Paeoniaceae Raf., Papaveraceae Juss., Passifloraceae Juss. *ex* Roussel, Paulowniaceae Nakai, Phrymaceae Schauer, Phytolaccaceae R.Br., Pinaceae Spreng. *ex* F.Rudolphi, Pittosporaceae R.Br., Plantaginaceae Juss., Platanaceae T.Lestib., Plumbaginaceae Juss., Poaceae, Podocarpaceae Endl., Polygalaceae Hoffmanns. & Link, Polygonaceae Juss., Polypodiaceae J.Presl & C.Presl, Pontederiaceae Kunth, Portulacaceae Juss., Posidoniaceae Vines, Potamogetonaceae Bercht. & J.Presl, Primulaceae Batsch *ex* Borkh., Proteaceae Juss., Pteridaceae E.D.M.Kirchner, Punicaceae Horan., Pyrolaceae Dumort., Rafflesiaceae Dumort., Ranunculaceae Juss., Resedaceae Martinov, Rhamnaceae Juss., Rosaceae Juss., Rubiaceae Juss., Rutaceae Juss., Salicaceae Mirb., Salviniaceae Martinov, Santalaceae R.Br., Sapindaceae Juss., Saxifragaceae Juss., Scrophulariaceae Juss., Selaginellaceae Willk., Simaroubaceae DC., Smilacaceae Vent., Solanaceae Juss., Sparganiaceae Hanin, Staphyleaceae Martinov, Sterculiaceae Vent., Tamaricaceae Link, Taxaceae Gray, Taxodiaceae Saporta, Theaceae Mirb. *ex* Ker Gawl., Thelypteridaceae Pic.Serm., Thymelaeaceae Juss., Tiliaceae Juss., Tropaeolaceae Juss. *ex* DC., Typhaceae Juss., Ulmaceae Mirb., Urticaceae Juss., Valerianaceae Batsch, Verbenaceae J.St.-Hil., Veronicaceae Cassel, Violaceae Batsch, Vitaceae Juss., Xanthorrhoeaceae Dumort., Zannichelliaceae Chevall., Zingiberaceae Martinov, Zygophyllaceae R.Br.
